# Impact of Micropolymorphism Outside the Peptide Binding Groove in the Clinically Relevant Allele HLA-C*14 on T Cell Responses in HIV-1 Infection

**DOI:** 10.1128/jvi.00432-22

**Published:** 2022-04-27

**Authors:** Takayuki Chikata, Wayne Paes, Nozomi Kuse, Thomas Partridge, Hiroyuki Gatanaga, Yu Zhang, Kimiko Kuroki, Katsumi Maenaka, Nicola Ternette, Shinichi Oka, Persephone Borrow, Masafumi Takiguchi

**Affiliations:** a Tokyo Laboratory and Division of International Collaboration Research, Joint Research Center for Human Retrovirus Infection, Kumamoto University, Kumamoto, Japan; b Nuffield Department of Clinical Medicine, University of Oxford, Oxford, United Kingdom; c AIDS Clinical Center, National Center for Global Health and Medicine, Tokyo, Japan; d Laboratory of Biomolecular Science, Faculty of Pharmaceutical Sciences, Hokkaido University, Sapporo, Japan; Emory University

**Keywords:** HLA-C, HIV-1, CD8^+^ T cells, epitopes, LC-MS/MS, peptides

## Abstract

There is increasing evidence for the importance of human leukocyte antigen C (HLA-C)-restricted CD8^+^ T cells in HIV-1 control, but these responses are relatively poorly investigated. The number of HLA-C-restricted HIV-1 epitopes identified is much smaller than those of HLA-A-restricted or HLA-B-restricted ones. Here, we utilized a mass spectrometry-based approach to identify HIV-1 peptides presented by HLA-C*14:03 protective and HLA-C*14:02 nonprotective alleles. We identified 25 8- to 11-mer HLA-I-bound HIV-1 peptides from HIV-1-infected HLA-C*14:02^+^/14:03^+^ cells. Analysis of T cell responses to these peptides identified novel 6 T cell epitopes targeted in HIV-1-infected HLA-C*14:02^+^/14:03^+^ subjects. Analyses using HLA stabilization assays demonstrated that all 6 epitope peptides exhibited higher binding to and greater cell surface stabilization of HLA-C*14:02 than HLA-C*14:03. T cell response magnitudes were typically higher in HLA-C*14:02^+^ than HLA-C*14:03^+^ individuals, with responses to the Pol KM9 and Nef epitopes being significantly higher. The results show that HLA-C*14:02 can elicit stronger T cell responses to HIV-1 than HLA-C*14:03 and suggest that the single amino acid difference between these HLA-C14 subtypes at position 21, outside the peptide-binding groove, indirectly influences the stability of peptide-HLA-C*14 complexes and induction/expansion of HIV-specific T cells. Taken together with a previous finding that KIR2DL2^+^ NK cells recognized HLA-C*14:03^+^ HIV-1-infected cells more than HLA-C*14:02^+^ ones, the present study indicates that these HLA-C*14 subtypes differentially impact HIV-1 control by T cells and NK cells.

**IMPORTANCE** Some human leukocyte antigen (HLA) class I alleles are associated with good clinical outcomes in HIV-1 infection and are called protective HLA alleles. Identification of T cell epitopes restricted by protective HLA alleles can give important insight into virus-immune system interactions and inform design of immune-based prophylactic/therapeutic strategies. Although epitopes restricted by many protective HLA-A/B alleles have been identified, protective HLA-C alleles are relatively understudied. Here, we identified 6 novel T cell epitopes presented by both HLA-C*14:02 (no association with protection) and HLA-C*14:03 (protective) using a mass spectrometry-based immunopeptidome profiling approach. We found that these peptides bound to and stabilized HLA-C*14:02 better than HLA-C*14:03 and observed differences in induction/expansion of epitope-specific T cell responses in HIV-infected HLA-C*14:02^+^ versus HLA-C*14:03^+^ individuals. These results enhance understanding of how the microstructural difference at position 21 between these HLA-C*14 subtypes may influence cellular immune responses involved in viral control in HIV-1 infection.

## INTRODUCTION

Numerous studies have documented associations between human leukocyte antigen (HLA) class I alleles and clinical outcome in HIV-1 infection (1–11). Most of these studies focused on HLA-A/B alleles, and the role of HLA-C alleles in HIV-1 infection has been less thoroughly explored. A previous study showed that HLA-C expression level as a continuous variable correlated inversely with HIV-1 viral load and that there was a strong positive correlation between HLA-C expression level and HIV-specific T cell responses, suggesting that HIV-1 replication was suppressed by T cell-mediated immune pressure induced by higher expression of HLA-C alleles (12). In addition, previous studies of HLA class I alleles associated with clinical outcomes in antiretroviral therapy (ART)-naive Japanese individuals living with HIV-1 subtype B and in ART-naive Vietnamese cohorts living with HIV-1 subtype A/E showed that HLA-C*12:02 was a protective allele in HIV-1 infection (10, 11), although there is a strong linkage disequilibrium between HLA-C*12:02 and HLA-B*52:01, also a protective allele in Japan (10). Subsequent studies clarified that HLA-C*12:02-restricted T cells specific for two epitopes effectively suppressed HIV-1 replication in ART-naive Japanese individuals (13). Furthermore, our recent studies showed that an HLA-A*29:01-B*07:05-C*15:05 haplotype was associated with poor clinical outcome in Vietnam (11) and subsequently demonstrated that its detrimental effect was due to the accumulation of escape mutations selected by HLA-C*15:05-restricted cytotoxic T lymphocytes (CTLs) (14). Together, these studies indicate that HLA-C also plays an important role in control of HIV-1 infection.

A previous study of HLA alleles associated with clinical outcome in treatment-naive Japanese individuals living with HIV-1 showed that subjects with HLA-C*14:03 exhibited the lowest plasma viral loads (pVL) among groups of individuals with each of the HLA-C alleles examined and that HLA-C*14:03 was significantly associated with low pVL, whereas HLA-C*14:02 was not (10). Subsequent analysis demonstrated the influence of KIR alleles on this association, as HLA-C*14:03^+^ KIR-2DL2/2DS2^+^ individuals had significantly lower pVL than those with either HLA-C*14:03 or KIR-2DL2/2DS2, while KIR-2DL2/2DS2 did not influence pVL in HLA-C*14:02^+^ individuals (15). These findings suggested that KIR-2DL2/2DS2^+^ NK cells contribute to control of viremia in HLA-C*14:03^+^ individuals. However, as HLA-C*14:02- and HLA-C*14:03-restricted HIV-1 epitopes have not yet been thoroughly identified, whether there are any differences in the HIV-1-specific T cell responses induced in individuals with each of these HLA-C*14 alleles and the contribution made by these T cell responses to clinical outcome remain unknown.

HLA class I-restricted HIV-1 epitopes have been identified using a number of strategies, including systematic screening of overlapping peptides spanning one or more viral proteins and/or *in silico* prediction of peptides likely to bind to particular HLA alleles. Although there are over 250 optimal HIV-1 CTL epitopes listed in the LANL database of HIV-1 epitopes that have been defined to date (LANL-HSD [www.hiv.lanl.gov]), only one HLA-C*14-restricted HIV-1 epitope is reported in this database. Mass spectrometry (MS)-based approaches have also been developed for identification of T cell epitopes and have been employed in several recent infectious disease and cancer studies (16–27). Application of MS-based methods for profiling of peptides presented on HIV-1-infected cells expressing a single HLA allele has been shown to be an effective method for identifying epitopes restricted by a given HLA allele (25, 28). As there has historically been a paucity of data available to inform the development of good algorithms for *in silico* prediction of HLA-C-restricted epitopes, MS-based methods offer tremendous advantages for identification of HLA-C-restricted epitopes. Notably, in a previous study, we were able to identify novel HLA-C*12:02-restricted immunodominant HIV-1 epitopes by employing an MS-based immunopeptidome profiling approach (28).

Here, we first sought to identify HIV-1 peptides bound to two HLA-C*14 subtypes, HLA-C*14:02 and HLA-C*14:03, by using liquid chromatography-tandem mass spectrometry (LC-MS/MS). Following verification of HLA-C*14:02/C*14:03 binding using an HLA stabilization-based peptide binding method, we screened peptides for T cell recognition in *ex vivo* enzyme-linked immunospot (ELISPOT) assays to identify HLA-C*14:02/C*14:03-restricted T cell epitopes. We subsequently investigated the ability of epitope-specific HLA-C*14:02/C*14:03-restricted T cells to recognize HLA-C*14:02^+^ or HLA-C*14:03^+^ target cells and compared induction of T cell responses to that seen with the epitopes identified in HIV-1-infected HLA-C*14:02^+^ and HLA-C*14:03^+^ individuals. Our study identified six HLA-C*14:02/C*14:03-restricted epitopes targeted in HIV-infected individuals, highlighting the efficacy of LC-MS/MS-based methods for identification of HLA-C-restricted T cell epitopes. Importantly, our findings also suggest that the single amino acid difference in the closely related HLA-C*14:02/C*14:03 alleles, which is located at position 21, outside the floor of the peptide-binding groove, influences peptide-HLA-C*14 complex stability and induction of HIV-1-specific T cells.

## RESULTS

### Identification of HLA-C*14:02- and HLA-C*14:03-binding HIV-1 peptides by LC-MS/MS.

**FIG 1:**
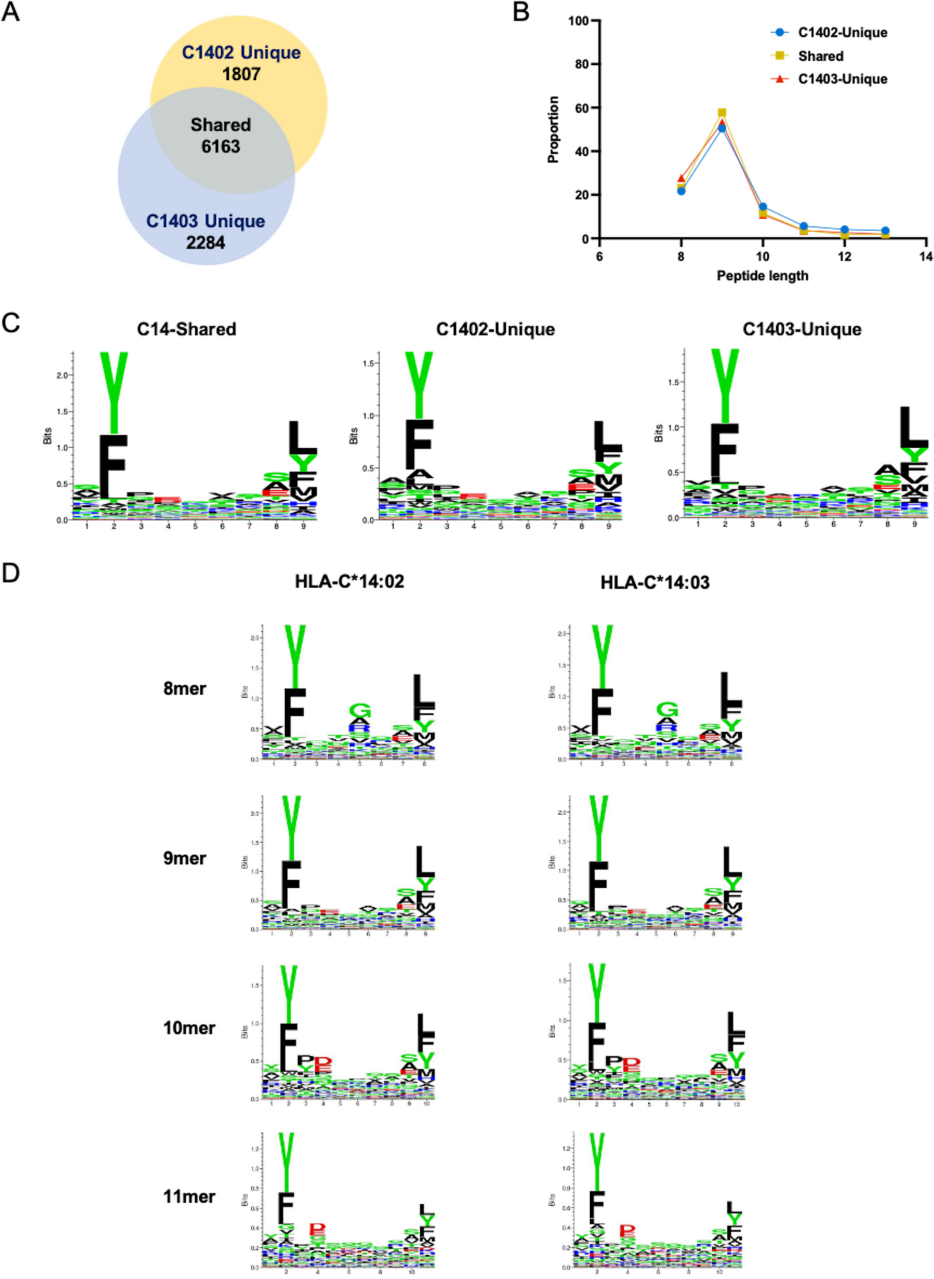
Characteristics of HLA-C*14:02 and C*14:03-bound peptides identified by LC-MS/MS. (A) Number of unique peptides identified in HIV-1 NL4-3-infected .221-C1402 and .221-C1403 cells. The frequency of p24^+^ cells in both NL4-3-infected .221-C1402 cells and NL4-3-infected .221-C1403 cells was 50 to 60%. The numerical overlap in peptide identification between HLA-C*14:02 and -C*14:03 is displayed in area-proportional Venn diagrams. (B) Length distribution of 8- to 13-mer peptides eluted from NL4-3-infected .221-C1402 or NL4-3-infected .221-C1403 cells. (C) Sequence logos of 9-mer peptides shared in the data sets for both alleles and 9-mer peptides detected only in eluates from either NL4-3-infected .221-C1402 or NL4-3-infected .221-C1403 cells. (D) Sequence logos of all 8- to 11-mer peptides in eluates from NL4-3-infected .221-C1402 or .221-C1403 cells.

721.221 cells expressing CD4 and HLA-C*14:02 (.221-C1402) or HLA-C*14:03 (.221-C1403) were infected with the laboratory-adapted NL4-3 HIV-1 strain. HLA-peptide complexes were isolated from cell lysates by immunoprecipitation with the anti-HLA class I antibody W6/32; then, peptides were eluted, fractionated by high-performance liquid chromatography (HPLC), and finally analyzed by LC-MS/MS. Two independent replicate experiments were performed, in which a total of 7,970 and 8,447 unique 8- to 13-mer peptide sequences were identified from .221-C1402 and .221-C1403 cells, respectively (Fig. 1A). Of these peptides, 6,163 were identified in both the C*14:02- and C*14:03-bound peptide data sets, while other peptides were detected in the acid-eluted fraction from only a single allele. The proportion of peptides identified in LC-MS/MS runs performed with only one of the two alleles did not exceed that observed in the replicate analyses performed with each individual allele or in biological replicate experiments we conducted previously (25), indicative of a high degree of concordance in the peptides bound by each allele. Analysis of the length distribution of 8- to 13-mer peptides eluted from C*14:02/C*14:03 indicated a typical HLA class I-restricted profile and revealed no substantial differences between the two alleles in peptide length preferences (Fig. 1B). Moreover, no noteworthy differences were observed in the binding motifs of the 9-mer peptides shared in both the C*14:02 and C*14:03 data sets and peptides detected only in LC-MS/MS analyses of eluates from either C*14:02 or C*14:03 (Fig. 1C), providing further evidence to suggest that very similar peptides are bound by both alleles. Analysis of the peptide motifs of all 8-, 9-, 10- and 11-mer peptides (the lengths of most of reported CTL epitopes) bound to each allele revealed a preference for a tyrosine and phenylalanine residue at the second position and a preference for hydrophobic residues, including leucine, tyrosine, phenylalanine, and methionine, at the C terminus (Fig. 1D). These motifs are consistent with the HLA-C*1402/03 peptide motifs previously estimated using reported epitopes (29).

A total of 31 unique HIV-1 NL4-3-derived peptide sequences were detected in the four experiments, 6 of which were background peptides routinely copurified with HLA-I immunoprecipitates from HIV-1-infected .221 cells (25), while 25 were anticipated to be HLA-C*14:02/03 bound (Table 1). Sixteen of the latter were detected in samples from HIV-1-infected .221-C1402 cells, while 22 were detected from HIV-1 infected .221-C1403 cells. One of these peptides, Gag LL8 (LYNTIAVL), was a sequence variant of Gag LL8-5V7T (LYNTVATL), a peptide that was previously shown to be an HLA-C14-restricted HIV-1 T cell epitope (30), whereas the other 24 peptides were not described as HLA-C14-restrcted epitopes. We reported some of these HLA-C*14:02/C*14:03-derived HIV-1 peptides in a prior publication in which we employed data sets from multiple immunopeptidome profiling experiments to enable calculation of the relative proportions of HIV-derived conventional versus spliced HLA-I-bound peptides (see the supplemental material in reference 31).

**TABLE 1:** HIV-1 NL4-3-derived HLA-bound peptides identified from HIV-1-infected HLA-C*14:02- and HLA-C*14:03-expressing cells

Eluted NL4-3 peptide	Name	Length	C1402^*a*^	C1403	Protein and position
LYNTIAVL	Gag LL8	8	●	●	Gag 78–85
LYNTIAVLY	Gag LY9	9	●	●	Gag 78–86
AFSPEVIPM	Gag AM9	9	●		Gag 163–171
FFREDLAF	Pol FF8	8		●	Pol 1–8
AFPQGKAREF	Pol AF10	10	●	●	Pol 7–16
PQGKAREF	Pol PF8	8		●	Pol 9–16
VWGRDNNSL	Pol VWL9	9	●	●	Pol 33–41
VYYDPSKDL	Pol VYL9	9	●	●	Pol 472–480
VYYDPSKDLIA	Pol VA11	11		●	Pol 472–482
FYVDGAANRET	Pol FT11	11		●	Pol 595–605
VYLAWVPAH	Pol VH9	9		●	Pol 686–694
KYHSNWRAM	Pol KM9	9	●	●	Pol 729–737
ASGYIEAEV	Pol AV9	9		●	Pol 795–803
FMTKALGI	Tat FI8	8		●	Tat 38–45
SFNISTSI	Env SI8	8		●	Env 158–165
AFVTIGKIGNM	Env AM11	11		●	Env 316–326
VTIGKIGNM	Env VM9	9	●		Env 318–326
FLGAAGSTM	Env FM9	9	●	●	Env 522–530
YTSLIHSLI	Env YI9	9	●		Env 638–646
IYHTQGYF	Nef IF8	8	●	●	Nef 114–121
IYHTQGYFP	Nef IP9	9	●	●	Nef 114–122
YFPDWQNYT	Nef YT9	9	●	●	Nef 120–128
RFDSRLAF	Nef RF8	8	●	●	Nef 184–191
AFHHVAREL	Nef AL9	9	●	●	Nef 190–198
FHHVAREL	Nef FL8	8	●	●	Nef 191–198

a Closed circles indicate the peptide which was identified in HLA-C*1402 or/and -C*1403 expressing cells.

### Binding of the eluted HIV-1 peptides to HLA-C*14:02 and C*14:03.

**FIG 2:**
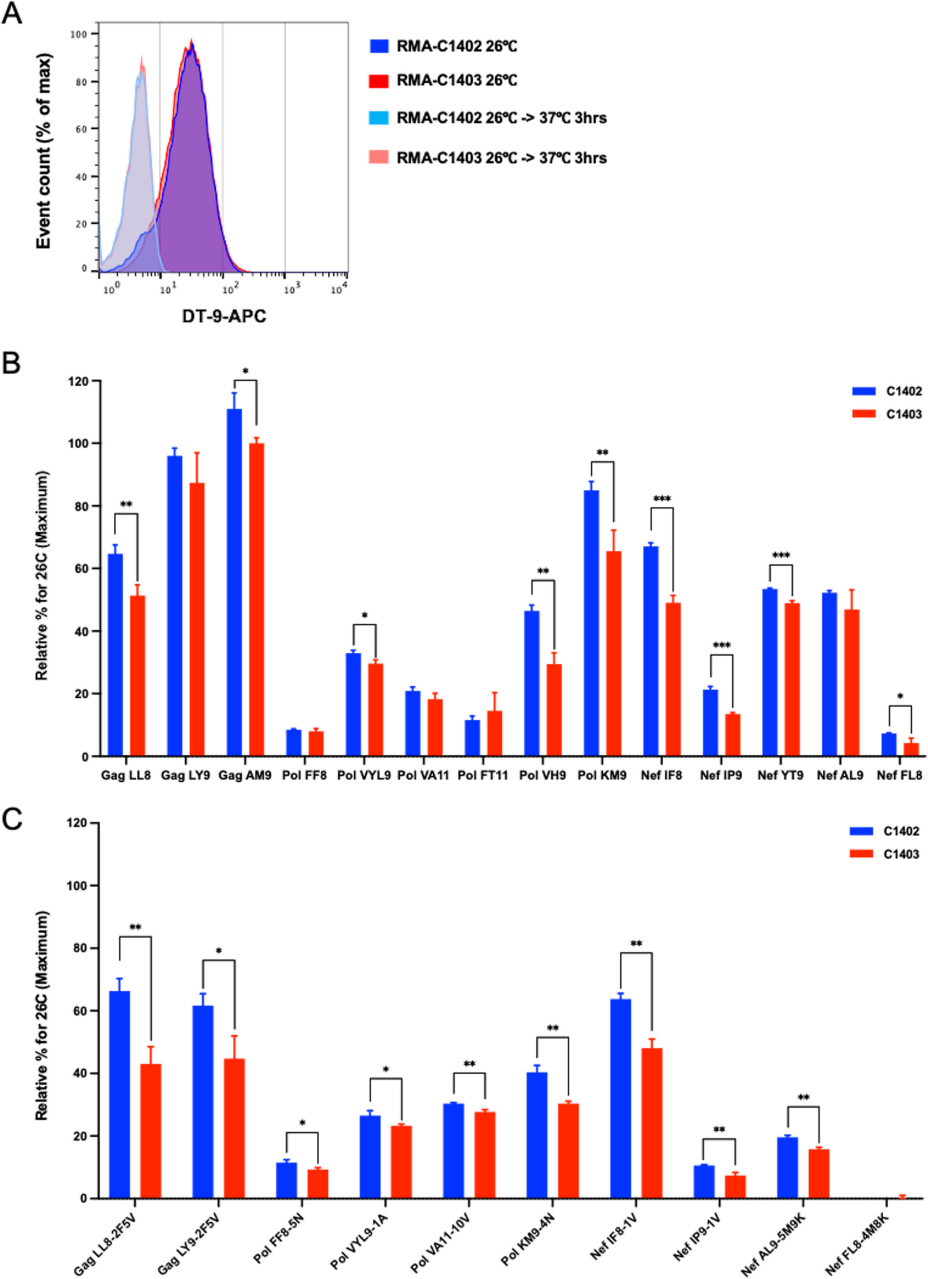
HLA-C*14:02/14:03 binding of HIV-1 peptides eluted from NL4-3-infected .221-C1402 and .221-C1403 cells as assessed by HLA stabilization assay using RMA-S cell lines. (A) Histogram plots showing surface expression of HLA-C*14:02 or HLA-C*14:03 on RMA-S-C1402 or RMA-S-C1403 cells at 26°C and after cells cultured at 26°C had been warmed to 37°C for 3 h. MFI values of cells cultured at 26°C were 27.4 for HLA-C*14:02 and 28.1 for HLA-C*14:03. MFI values of cells cultured at 37°C for 3 h were 5.0 for HLA-C*14:02 and 6.54 for HLA-C*14:03. (B) Analysis of binding of 14 HIV-1 NL4.3 peptides identified by LC-MS/MS to HLA-C*14:02 (blue) or HLA-C*14:03 (red) as measured in HLA class I stabilization assays using RMA-S-C1402 or RMA-S-C1403 cells. (C) Analysis of binding of 9 additional variant peptides to HLA-C*14:02 (blue) or C*14:03 (red). For panels B and C, peptides were tested at a concentration of 100 μM. Results are plotted as percent surface HLA-I expression relative to that at 26°C, calculated as the ratio of the MFI of peptide-pulsed RMA-S-C1402 or RMA-S-C1403 cells to that of control (non-peptide-pulsed) cells kept at 26°C, multiplied by 100. Means and standard deviations (SDs) from triplicate assays are shown. Statistical analyses were performed by unpaired *t* test. The *P* values were corrected for multiple testing. *, *P* < 0.05 and *q *< 0.05; **, *P < *0.01 and *q *< 0.03; ***, *P < *0.001 and *q *< 0.005.

We next sought to confirm the HLA-C*14:02/C*14:03 restriction of the HIV-1 peptides identified by LC-MS/MS. First, we tested their ability to bind to HLA-C*14:02 or HLA-C*14:03 using HLA stabilization assays. Of the 25 HIV-1 peptides, three Env peptides, Env SI8, Env AM11, and Env VM9, are located in variable regions V2 and V3. In addition, Pol AF10, Pol PF8, Pol VWL9, and Env YI9 are located in regions where high sequence variation is reported in the LANL database. Therefore, we excluded these peptides from further analysis. The other 18 peptides were synthesized and tested for binding to HLA-C*14:02/C*14:03 by analyzing their ability to stabilize surface HLA expression on TAP-defective RMA-S cell lines expressing HLA-C*14:02 (RMA-S-C1402) or HLA-C*14:03 (RMA-S-C1403). These RMA-S cell lines express equivalent levels of HLA-C*14:02 and HLA-C*14:03 on the cell surface at 26°C, and surface HLA expression declines to very low levels when the cells are warmed to 37°C in the absence of exogenously added stabilizing peptides (Fig. 2A). Peptide binding was determined by evaluating whether HLA-I expression was stabilized in the presence of 100 μM peptide. Fourteen of 18 peptides were shown to bind to both HLA-C*14 molecules using this approach (Fig. 2B). However, 4 peptides (Pol AV9, Tat FI8, Env FM8, and Nef RF8) did not exhibit detectable binding to the HLA-C*14 molecules in this assay. In a previous study in which we sought to identify HLA-C*12:02-restricted HIV-1 epitopes, some of the peptides identified using LC-MS/MS that did not show binding to HLA-C*12:02 in the HLA stabilization assay could be refolded with HLA-C*12:02 heavy chain and β_2_m molecules in a refolding assay, but T cells specific for these low-affinity peptides were not observed in an *ex vivo* ELISPOT assay, suggesting that they did not elicit strong T cell responses in natural infection (28). Therefore, we did not include these 4 peptides in further experiments.

Next, we sought to determine the impact of differences between NL4-3 peptide sequences and those of viral variants in a Japanese cohort on peptide binding to HLA-C*1402/03. We first exploited preexisting HIV-1 sequence data from our chronically HIV-1 subtype B-infected Japanese cohort to identify peptide sequence variants present at frequencies of >10% in this cohort (Table 2). Of the 14 peptides studied, 11 peptides had one or more variant sequences (total *n* = 16) present at frequencies above 10% in our cohort. We previously identified 3 adaptive HLA-C*14:02- and/or HLA-C*14:03-associated mutations, TGag81A, YNef120F, and QNef125D (32). It is assumed that the 6 peptides including one of these mutations may be escape mutant epitopes. As escape mutant-specific T cells are rarely elicited in HIV-1-infected individuals, we excluded these peptides from further analysis. We synthesized the other 10 peptides and then tested binding of these peptides to HLA-C*14:02 and -C*14:03 molecules at a concentration of 100 μM by performing HLA stabilization assays. All 10 peptides exhibited detectable binding to the HLA-C*14 molecules with the exception of NefFL8-4M8K (Fig. 2C).

**TABLE 2:** Peptide sequence variants present in >10% of patient viruses in the chronically HIV-1-infected Japanese cohort

Eluted NL4-3 peptide	Name	Frequency	Mutant 1	Frequency	Mutant 2	Frequency
LYNTIAVL	Gag LL8	4.3	LFNTVAVL ^*a*^	12.6	LFNAVAVL	10.6
LYNTIAVLY	Gag LY9	4.3	LFNTVAVLY ^*a*^	12.1	LFNAVAVLY	10.6
AFSPEVIPM ^*a*^	Gag AM9	84.6				
FFREDLAF	Pol FF8	14.9	FFRENLAF ^*a*^	71.6		
VYYDPSKDL ^*a*^	Pol VYL9	60.1	AYYDPSKDL	11.0		
VYYDPSKDLIA ^*a*^	Pol VA11	32.5	VYYDPSKDLVA	24.5		
FYVDGAANRET ^*a*^	Pol FT11	72.0				
VYLAWVPAH ^*a*^	Pol VH9	82.4				
KYHSNWRAM ^*a*^	Pol KM9	59.0	KYHNNWRAM	17.9		
IYHTQGYF	Nef IF8	3.9	VYHTQGYF	33.2	VYHTQGFF ^*a*^	35.5
IYHTQGYFP	Nef IP9	3.9	VYHTQGYFP	32.6	VYHTQGFFP ^*a*^	35.5
YFPDWQNYT ^*a*^	Nef YT9	26.1	FFPDWQNYT	19.4	FFPDWHNYT	10.3
AFHHVAREL	Nef AL9	3.5	AFHHMAREK ^*a*^	16.5		
FHHVAREL	Nef FL8	3.5	FHHMAREK ^*a*^	16.5		

a HIV-1 consensus sequence in Japanese cohort.

Notably, in the assays performed with the 23 HLA-C*14-binding peptides, the relative level of peptide-induced stabilization of surface HLA-C*14:02 expression was typically higher than that for HLA-C*14:03, with the difference reaching statistical significance for 18 of these peptides (Fig. 2B and C). These results indicate that the substitution at position 21 affects the affinity of peptide binding to HLA-C*14:02 and HLA-C*14:03 and/or that HLA-C*14:02 is more readily stabilized by peptide binding.

### T cell responses to HLA-C*14:02- and C*14:03-binding HIV-1 peptides.

**FIG 3:**
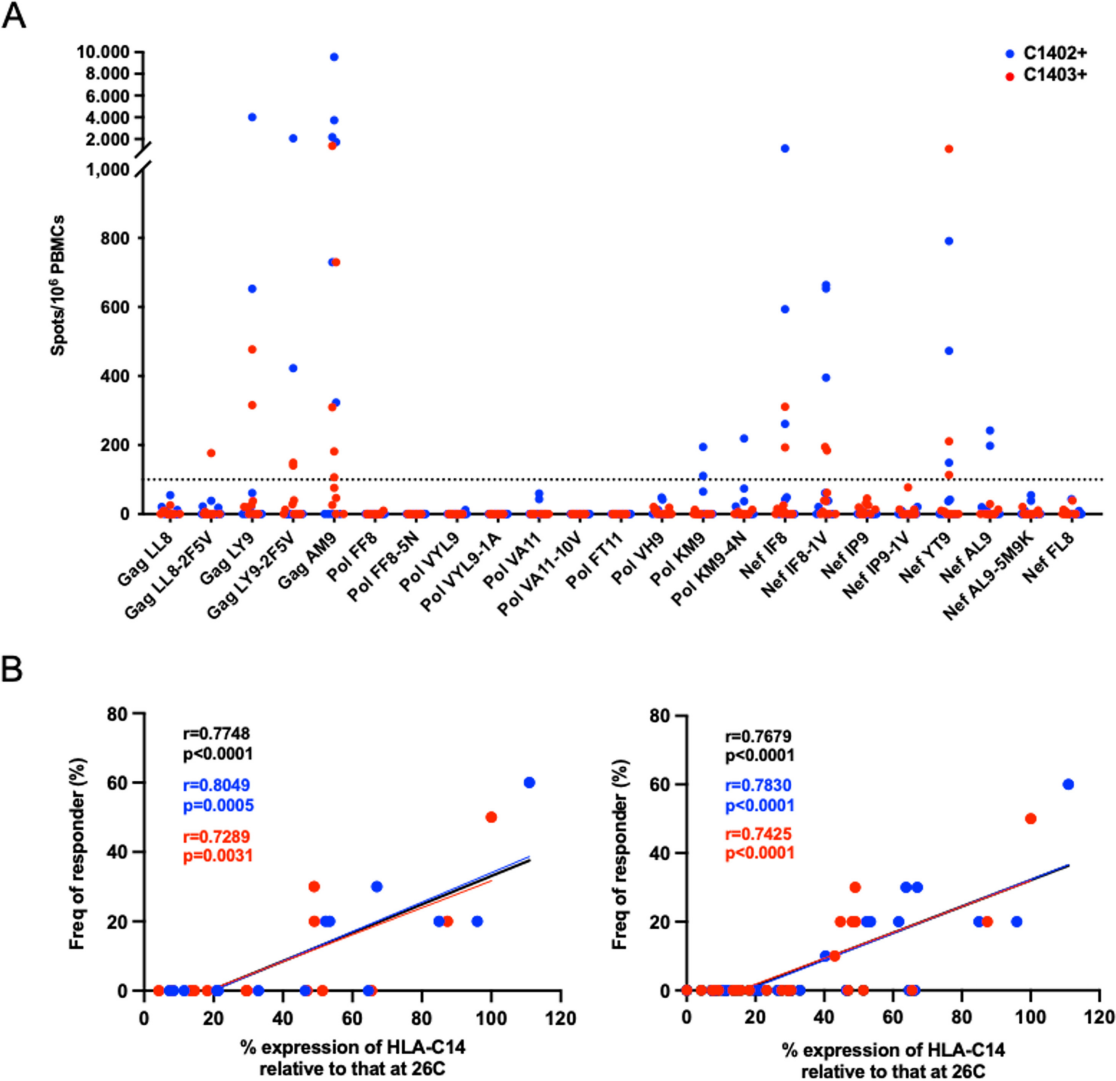
T cell responses to HLA-C*14:02- and/or HLA-C*14:03-binding HIV-1 peptides. (A) T cell responses to HLA-C*14:02/03-binding peptides in 20 chronically HIV-1-infected Japanese individuals with HLA-C*14:02 or HLA-C*14:03. T cell targeting of 14 putative epitopes was analyzed by testing responses to 14 NL4-3 sequence-based peptides and 9 peptides that were sequence variants thereof in *ex vivo* IFN-γ ELISPOT assays (peptides tested at a concentration of 1 μM). A positive response was defined as >100 spots/10^6^ PBMCs (indicated by the horizontal dotted line). (B) Correlation between peptide binding to HLA-C*14:02 or HLA-C*14:03 and the frequency of responders to these peptides in HLA-C*14:02^+^ (blue) and HLA-C*14:03^+^ (red) individuals for NL4-3 sequence-based versions of the peptides only (left) or both NL4-3 sequence-based and variant peptides (right). Statistical analyses were performed using Pearson's correlation test.

To clarify whether these HLA-C*14:02- and C*14:03-binding HIV-1 peptides were T cell epitopes, we investigated T cell responses to these peptides in HIV-1-infected individuals. We assessed T cell targeting of 14 putative HIV-1 epitope sites by testing responses to the 14 NL4-3 sequence-based peptides and 9 variant peptides in 10 HLA-C*14:02^+^ and 10 HLA-C*14:03^+^ treatment-naive individuals living with HIV-1 subtype B using *ex vivo* gamma interferon (IFN-γ) ELISPOT assays. To identify HLA-C*14-restricted immunodominant epitopes, we investigated whether these peptides were recognized by virus-specific T cells in 20 individuals enabling detection of epitopes typically targeted in >5% of HLA-C*14^+^ individuals. T cell responses to 7 target sites that were represented by 10 peptides (Gag LL8-2F5V, Gag LY9, Gag LY9-2F5V, Gag AM9, Pol KM9, Pol KM9-4N, Nef IF8, Nef IF8-1V, Nef YT9, and Nef AL9) were detected in one or more individuals (Fig. 3A). T cell responses to 6 peptides were found in both HLA-C*14:02^+^ and HLA-C*14:03^+^ individuals, whereas responses to 4 peptides (Gag LL8-2F5V, Pol KM9, Pol KM9-4N, and Nef AL9) were detected in only HLA-C*14:02^+^ or HLA-C*14:03^+^ individuals (Fig. 3A). No T cell recognition of Gag LL8 (LYNTIAVL), which differs at two amino acid positions from the published HLA-C14-restricted CTL epitope, Gag LL8-5V7T, was detected, and a variant of this peptide, Gag LL8-2F5V, was recognized in only one of the 20 individuals tested. However, responses were observed in 4 individuals to Gag LY9 (LYNTIAVLY), which includes the Gag LL8 peptide sequence. These findings suggest that the optimal epitope is the 9-mer sequence Gag LY9 (LYNTIAVLY) but not the 8-mer Gag LL8 (LYNTIAVL). T cell responses to variant versions of the Gag LY9, Pol KM9, and Nef IF8 peptides were detected in a number of individuals similar to the number with responses to their NL4-3 sequence-based counterparts (Fig. 3A). In contrast, although responses to the NL4-3 sequence version of the Nef AL9 peptide were observed in 2 individuals, responses to the variant peptide Nef AL9-5M9K were not detected in any of the 20 individuals studied. Together, these results suggest that at least 6 of the HLA-C*14-binding HIV-1 peptides identified by LC-MS/MS-based sequencing are novel epitopes targeted by T cell responses in HLA-C*14:02^+^ and/or HLA-C*14:03^+^ individuals.

We then investigated the correlation between the relative surface HLA-C*14 stabilization induced following binding of each peptide and the frequency of individuals exhibiting responses to the peptide in the HLA-C*14:02/C*14:03^+^ subjects tested. A strong positive correlation was observed between these parameters in analyses performed with just the NL4-3-derived peptides (Fig. 3B, left) and or both the NL4-3-derived and their variant peptides (Fig. 3B, right). The results also showed that no T cell responses to peptides which bound poorly and achieved less than 40% relative surface HLA-C*14 stabilization expression were detected, suggesting that more weakly binding peptides may not induce efficient epitope-specific T cell priming/expansion in individuals with HLA-C*14:02 or -C*14:03. Moreover, these findings support the idea that peptides with higher binding affinity are more likely to be targets for immunodominant T cell responses.

**FIG 4:**
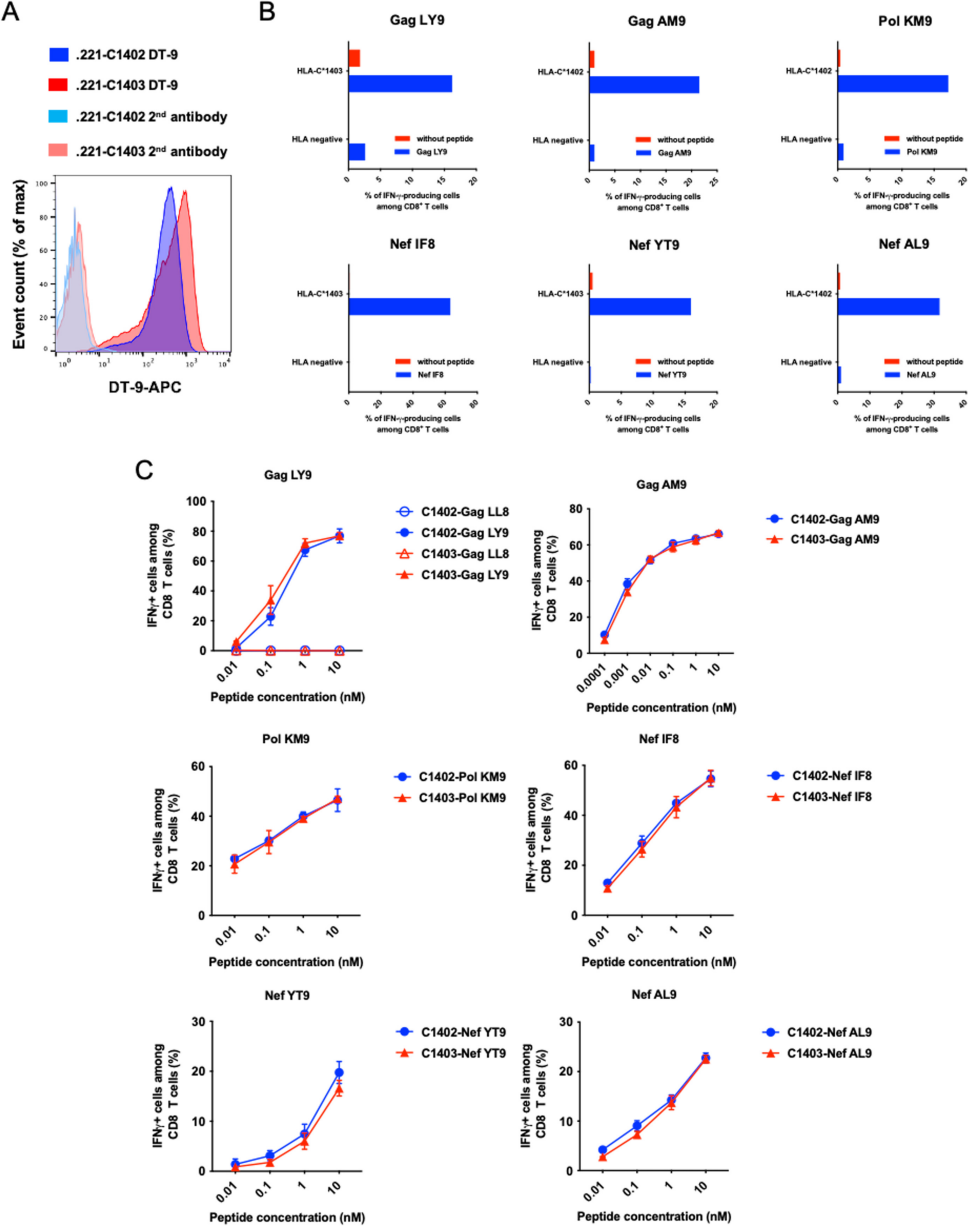
HLA-C*14:02- and HLA-C*14:03-restricted T cell responses to 6 putative epitope peptides. (A) Histogram plots showing surface expression of HLA-C*14:02 or HLA-C*14:03 on .221-C14:02 or .221-C14:03 cells. The relative expression level of HLA-C*14:02 versus HLA-C*14:03 [(MFI of .221-C1402 stained with DT9 − MFI of .221-C1402 stained with the second antibody)/(MFI of .221-C1403 stained with DT9 − MFI of .221-C1403 stained with the second antibody)] is 1.3. (B and C) Gag LY9-, Gag AM9-, Pol KM9-, Nef YT9-, Nef IF8-, and Nef AL9-specific bulk T cell populations were expanded by stimulating responders’ PBMCs with each peptide. The ability of these peptide-specific bulk T cells to recognize peptide-pulsed .221 cells expressing HLA-C*14 molecules was then analyzed by ICS assay. (B) T cell recognition of .221 cells expressing the patient’s autologous HLA-C*14 allele and non-HLA-C*14-expressing cells pulsed with 1 μM peptide. (C) T cell recognition of HLA-C*14:02 or HLA-C*14:03-expressing cells pulsed with the indicated peptides at concentrations from 0.01 to 10 nM was analyzed by an ICS assay. The results are means and SDs from triplicate assays.

To confirm the HLA-C*14-restriction of the T cell responses to the 6 NL4-3-derived peptides, we expanded T cells specific for these peptides by stimulating peripheral blood mononuclear cells (PBMCs) from responders with each peptide (HLA-C*14:02^+^ responders for Gag AM9, Pol KM9, and Nef AL9 and HLA-C*14:03^+^ responders for Gag LY9, Nef IF8, and Nef YT9). The cultured T cells were then tested for their ability to recognize the peptides presented on 721.221 cells expressing HLA-C*14:02 (.221-C1402 cells) or HLA-C*14:03 (.221-C1403 cells) and 721.221 cells by intracellular cytokine staining (ICS) assay. Both cell lines expressed similarly high levels of HLA-C*14, although the surface expression level of HLA-C*14 on the HLA-C*14:03-transfected line was higher than that on the HLA-C*14:02 line (the ratio of relative expression of HLA-C*14:03 to that of HLA-C*14:02 was approximately 1.3:1) (Fig. 4A). All of the T cells recognized the epitope peptide-pulsed .221 cells expressing their autologous HLA-C*14 allele (Fig. 4B), indicating that the T cell responses to each peptide were restricted by these HLA-C*14 molecules. We further analyzed cross-recognition of .221-C1402 and .221-C1403 cells prepulsed with peptides at a range of different concentrations by T cells from HLA-C*14:02^+^ and HLA-C*14:03^+^ individuals. No differences in T cell recognition of the peptide-pulsed .221-C1402 and .221-C1403 cells were observed (Fig. 4C), indicating that these T cells are capable of cross-recognizing the epitope peptides when presented by either of the two HLA-C*14 molecules.

### Presentation of HLA-C*14-restricted HIV-1 epitopes on HIV-1-infected cells.

**FIG 5:**
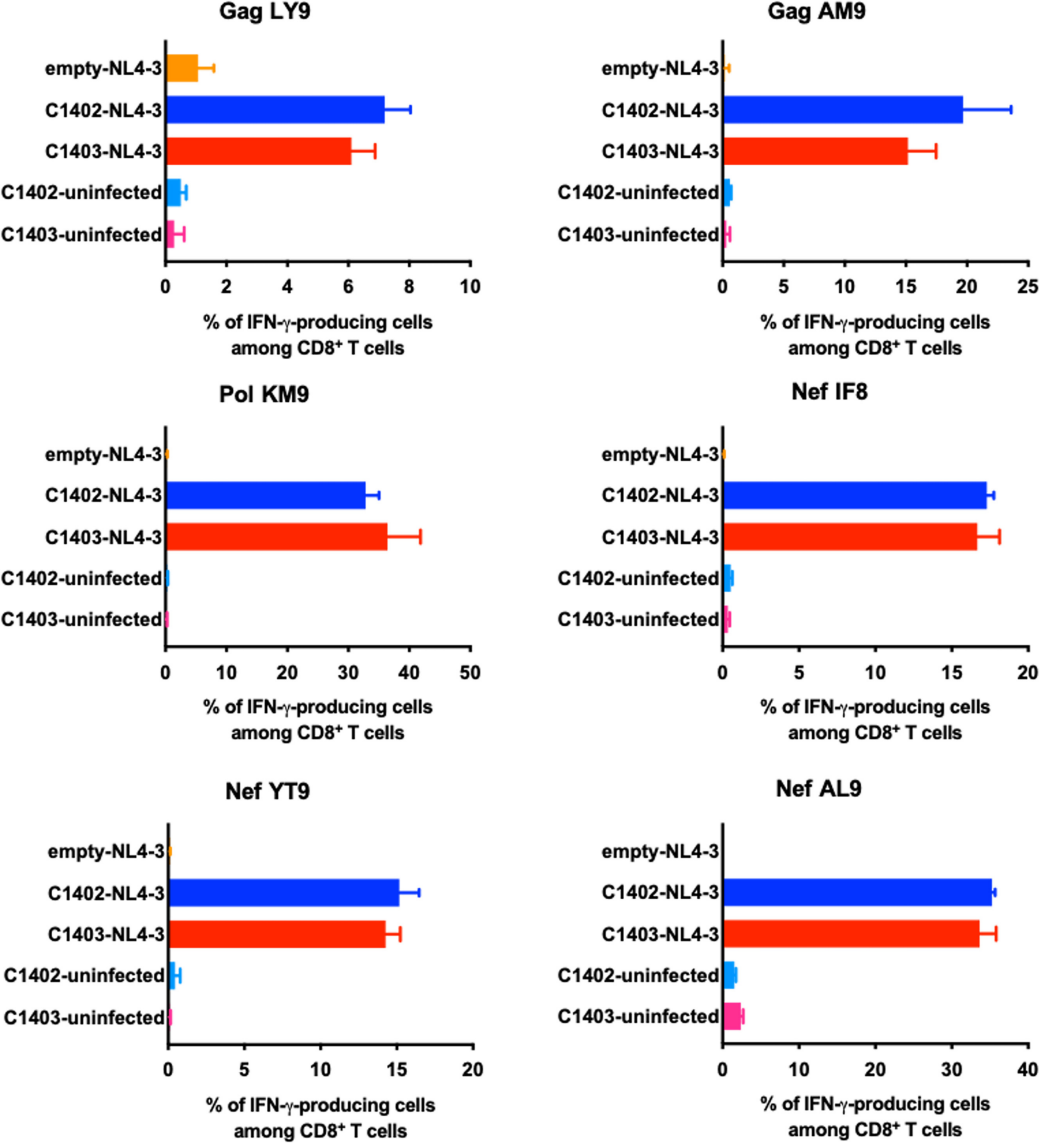
Recognition of NL4-3-infected .221-CD4 cells by peptide-specific CD8^+^ T cells. The response of bulk T cells specific for 6 peptides to NL4-3-infected .221-C1402 cells, NL4-3-infected .221-C1403 cells, NL4-3-infected 721.221 cells, uninfected .221-C1402 cells, and uninfected .221-C1403 cells was analyzed by ICS assay. The frequencies of p24^+^ cells were as follows: NL4-3-infected .221-C1402 cells, 52%; NL4-3-infected .221-C1403 cells, 54%. The results are means and SDs from triplicate assays.

We next investigated whether T cells specific for these peptides could recognize NL4-3-infected .221-C1402 or .221-C1403 cells. Strong functional activation of T cells recognizing all 6 epitopes was triggered by both NL4-3-infected .221-C1402 and NL4-3-infected .221-C1403 cells but not uninfected .221 cells (Fig. 5). These results indicate that Gag LY9, Gag AM9, Pol KM9, Nef YT9, Nef IF8, and Nef AL9 are novel HLA-C*14:02- and C*14:03-restricted epitopes which are effectively presented by HLA-C*14 molecules of both subtypes on HIV-1-infected cells. The results from *ex vivo* ELISPOT assays showed that the frequencies of HLA-C*14:02/C*14:03 individuals with detectable responses to these 6 peptides ranged from 10% to 55% (Fig. 3A). Taken together, these results suggest that these peptides are immunodominant T cell epitopes presented by HLA-C*14:02 and -C*14:03.

### Different effect of two HLA-C*14 subtype alleles on the epitope-specific T cell responses expanded in HIV-1 infection.

**FIG 6:**
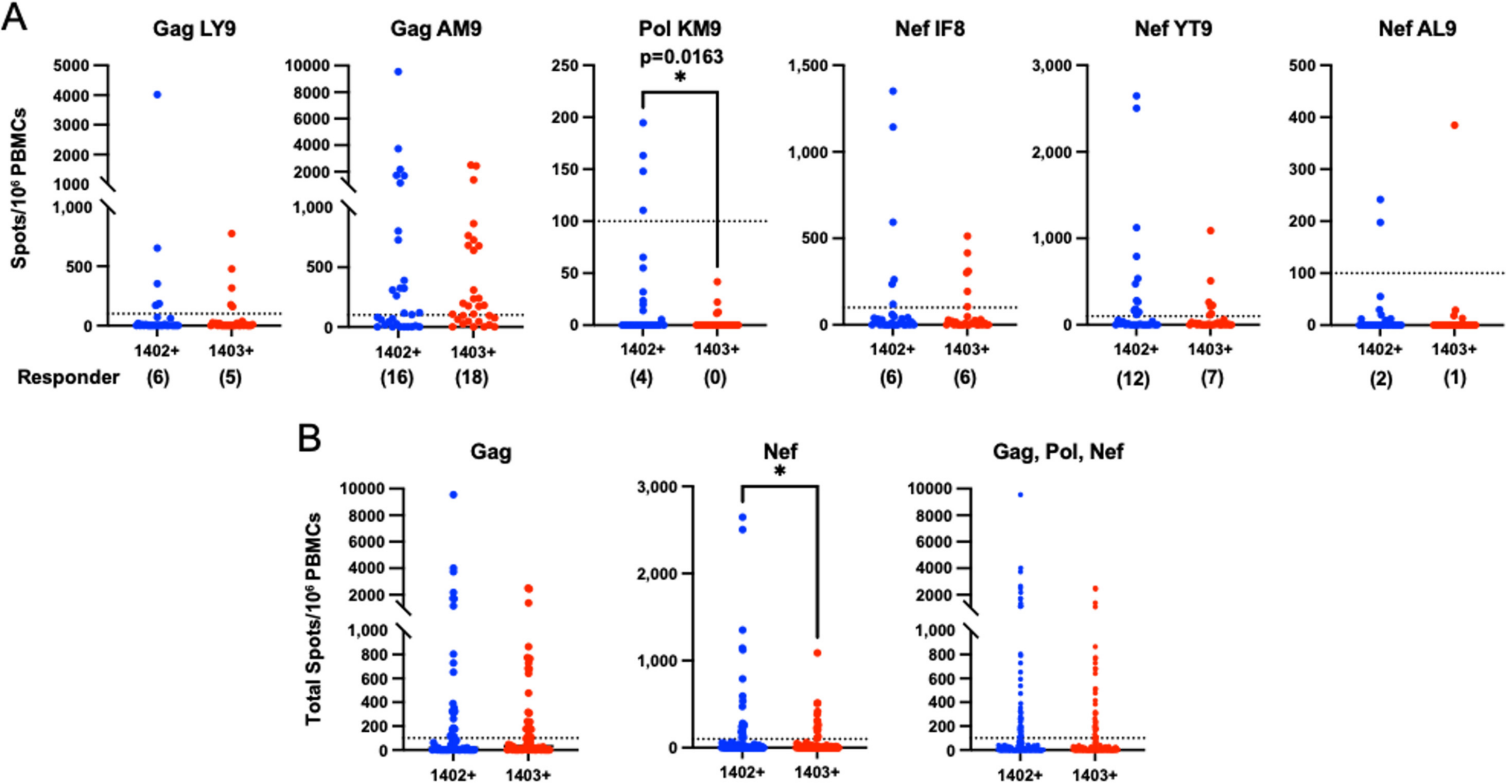
Difference in frequency of responses to HLA-C*14-restricted epitopes in HLA-C*14:02^+^ and HLA-C*14:03^+^ individuals. (A) T cell responses to 6 HLA-C*14:02/C*14:03 epitopes were analyzed in 30 HLA-C*14:02^+^ and 30 HLA-C*14:03^+^ treatment-naive individuals living with HIV-1. T cell responses to each peptide tested at a concentration of 1 μM were analyzed by *ex vivo* IFN-γ ELISPOT assay. Mean plasma viral load (log) and CD4 count in HLA-C*14:02^+^ or HLA-C*14:03^+^ individuals were 4.74 copies/mL and 337 cells/μL and 4.58 copies/mL and 333 cells/μL, respectively. A positive response was defined as >100 spots/10^6^ PBMCs (indicated by the horizontal dotted lines). Statistical analyses were performed by Mann-Whitney test. *, *P < *0.05. (B) T cell responses to Gag, Nef, and total epitopes. Statistical analyses were performed by Mann-Whitney test. *, *P < *0.05.

In our initial screening of HLA-C*14-binding peptides to identify those targeted by T cell responses, which employed samples from 10 HLA-C*14:02^+^ and 10 HLA-C*14:03^+^ individuals, responses to the 6 epitope peptides identified were detected in 10 to 55% of the 20 subjects tested, with T cell responses to Pol KM9 and Nef AL9 being observed only in HLA-C*14:02^+^ individuals (Fig. 3A). To confirm and extend our understanding of the proportion of individuals with T cell responses to these 6 epitope peptides, we analyzed responses to these epitopes in 40 additional HLA-C*14^+^ treatment-naive individuals (20 HLA-C*14:02^+^ and 20 HLA-C*14:03^+^). The T cell responses to these epitope peptides detected by *ex vivo* ELISPOT assay in the 60 HLA-C*14^+^ treatment-naive individuals tested together (30 HLA-C*14:02 and 30 HLA-C*14:03) are shown in Fig. 6A. The frequencies of individuals showing responses of >100 spot-forming cells/10^6^ PBMCs to these 6 epitopes in the 60 HLA-C*14:02/C*14:03 individuals tested were as follows: Gag LY9, 18%; Gag AM9, 56%; Pol KM9, 6.6%; Nef IF8, 20%; Nef YT9, 31%; and Nef AL9, 5%. The magnitude of T cell responses to Gag epitopes in responders was much higher than that of responses to Pol or Nef epitopes. Interestingly, the magnitude of T cell responses to Pol KM9 in HLA-C*14:02^+^ individuals was significantly higher than that in HLA-C*14:03^+^ individuals (Fig. 6A). Moreover, although the difference in response magnitude in subjects of each HLA-C*14 subtype did not reach statistical significance for any other individual epitope, the magnitude of T cell responses to Nef epitopes in HLA-C*14:02^+^ individuals was significantly higher than that in HLA-C*14:03^+^ individuals, and the magnitude of responses to all 6 epitopes showed a tendency to be higher in HLA-C*14:02^+^ than HLA-C*14:03^+^ individuals (Fig. 6B). Together, these results suggest that the single substitution at position 21 in these 2 HLA-C*14 subtypes affects the epitope-specific T cell responses expanded during HIV-1 infection.

### Relative binding affinity of 6 epitope peptides and comparison of peptide-induced HLA-C*14:02 versus HLA-C*14:03 stabilization.

**FIG 7:**
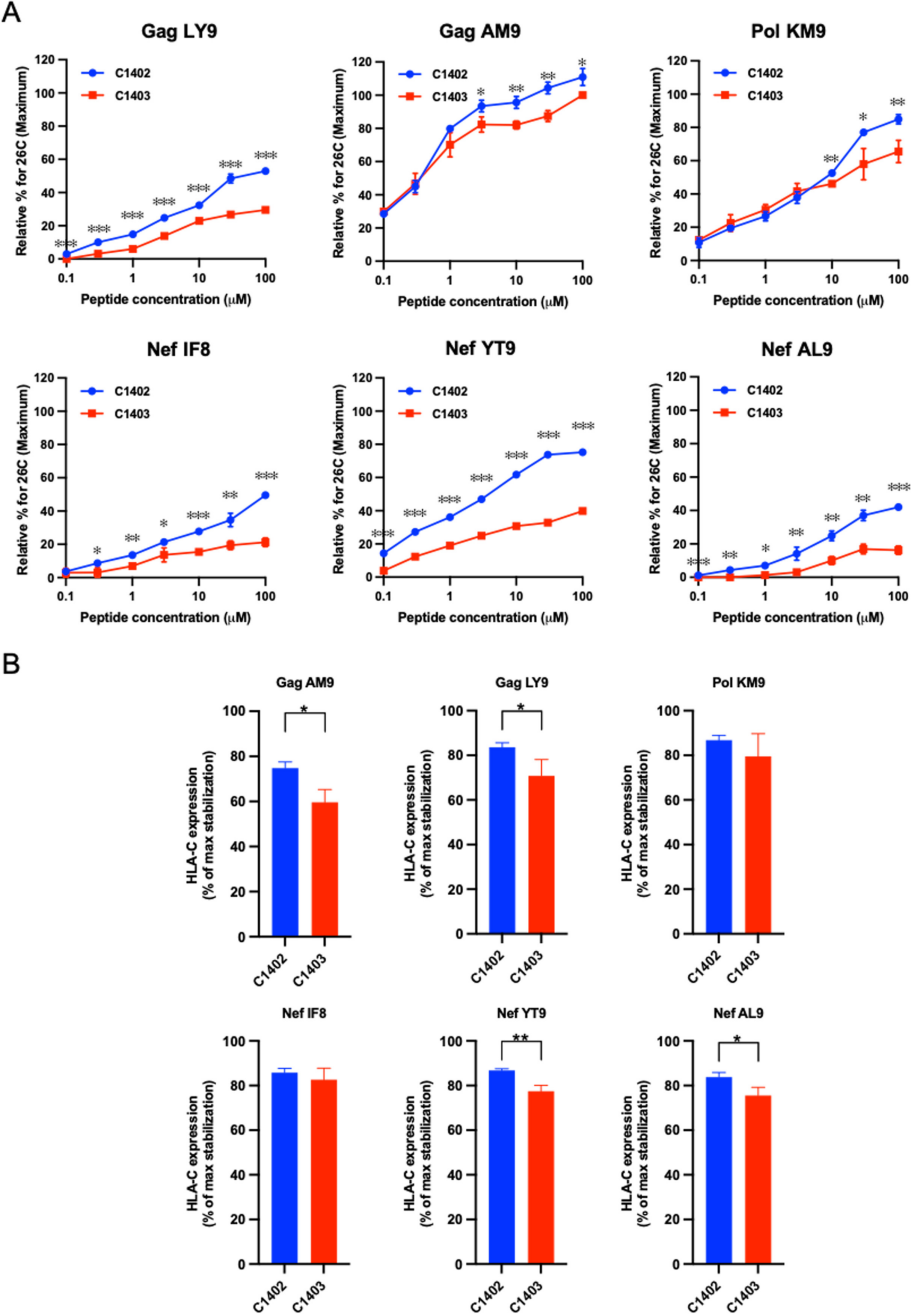
Peptide-induced stabilization of HLA-C*14:02 and C*14:03 molecules. (A) The relative affinity of binding of the 6 epitope peptides to HLA-C*14:02 (blue) or HLA-C*14:03 (red) was analyzed by testing HLA class I stabilization on RMA-S-C1402 or RMA-S-C1403 cells at peptide concentrations from 0.1 to 100 μM. Results are plotted as the percent increase in surface HLA-I expression relative to that at 26°C, calculated as the ratio of the MFI of peptide-pulsed RMA-S-C1402 or RMA-S-C1403 cells to that of control (non-peptide-pulsed) cells at 26°C multiplied by 100. Statistical analysis was performed by unpaired *t* test. *, *P < *0.05; **, *P < *0.01; ***, *P < *0.001. (B) The surface stability of HLA-C14-peptide complexes was determined using RMA-S-C1402 (blue) and RMA-S-C1403 (red) cells. The cells were incubated at 26°C with a 100 μM concentration of each of the 6 epitope peptides for 1 h and then warmed to 37°C for 3 h. After the excess external peptide had been washed off, cells were collected at 1 h. The relative MFI of HLA-C staining was plotted as the percentage of the maximal stabilization induced by peptide binding. Statistical analysis was performed by unpaired *t* test. The *P* values were corrected for multiple testing. *, *P < *0.05 and *q *< 0.1; **, *P < *0.01 and *q *< 0.05.

We found differences in the ability of many peptides to bind to and stabilize surface expression of HLA-C*14:02 and HLA-C*14:03 in HLA-C*14 stabilization experiments performed with 100 μM peptide (Fig. 2B). To clarify the relative binding affinity of 6 HLA-C*14:02/C*14:03-restricted epitope peptides for both HLA-C*14 subtypes, we performed HLA-C*14 stabilization experiments using a series of peptide concentrations. The results revealed a hierarchy of peptide-HLA-C*14 binding (with the Gag AM9 showing the highest and Nef AL9 the lowest relative affinity), and statistically significant differences were observed in the ability of all 6 peptides to induce stabilization of the two HLA-C*14 subtypes (Fig. 7A), confirming the difference in the affinity of peptide binding to the two subtypes. We further analyzed the stability of these peptide-HLA*C14 complexes using peptide-pulsed RMA-S-C*14 cells. RMA-S-C*1402 and -C*1403 cells were pulsed with epitope peptides at 26°C for 1 h and incubated at 37°C for 3 h in the presence of excess peptide; then, the cells were washed to remove free peptide, and the surface expression of HLA-C*14 at 37°C was measured 1 h later. A smaller decline in surface HLA expression was observed on peptide-pulsed HLA-C*1402 than HLA-C*1403-expressing RMA-S cells, with the difference reaching statistical significance for three of the peptides tested (Fig. 7B), indicative of a difference in the stability of the peptide–HLA-C*14 complexes formed by these subtypes. From these findings, it is speculated that the differences in relative affinity and stability of the peptide-HLA complexes formed by these two HLA-C*14 molecules influence induction and subsequent amplification of peptide-specific T cell responses, with a particularly pronounced effect being observed on subdominant responses such as that to the Pol and Nef epitopes.

## DISCUSSION

As HLA-C is expressed at lower levels on the cell surface than HLA-A or -B (33–35), it was originally assumed that HLA-C may make a less important contribution to control of HIV-1 infection. However, there is now increasing evidence to suggest the importance of HLA-C in HIV-1 infection. A large-scale study in an American cohort demonstrated that surface expression of HLA-C was correlated with HIV-1-specific T cell responses and viremia control in individuals living with HIV-1 (12). This study also showed strong T cell-mediated immune responses to HIV-1 epitopes presented by HLA-C alleles highly expressed on the cell surface, indicating that highly expressed HLA-C alleles may contribute to HIV-1 containment. Furthermore, other work showed that HLA-C*12:02 was significantly associated with low pVL and high CD4 count and that HLA-C*12:02-restricted T cells effectively suppressed HIV-1 replication in HIV-1-infected Japanese individuals (10). Nonetheless, although these studies support the idea that HLA-C-restricted T cells play an important role in clinical outcome of HIV-1 infection, HLA-C remains much less well studied than HLA-A and -B, and the number of HLA-C-restricted HIV-1 epitopes that have been identified is limited. We previously sought to identify T cell epitopes restricted by a protective allele, HLA-C*12:02, using an LC-MS/MS-based immunopeptidomics approach (28). In this study, we succeeded in detecting 2 previously reported and one novel immunodominant epitope among the peptides eluted from HLA-I molecules isolated from an NL4-3-infected .221-CD4 cell line expressing HLA-C*12:02. In the current study, we employed the same approach to analyze HIV-1-derived peptides in HLA-I eluates from HIV-1-infected cells expressing HLA-C*14:02 or HLA-C*14:03 and were able to identify 6 novel HLA-C*14-restricted epitopes. This provides further evidence that HLA-C -restricted T cell epitopes can be efficiently identified by LC-MS/MS-based immunopeptidome profiling.

The relative affinity of binding to HLA-C*1402/03 of 18 of the HIV-1 peptides identified in our immunopeptidome analysis was determined by assessing HLA stabilization assay on HLA-C*14-expressing RMA-S cell lines. Four of the peptides tested did not induce detectable stabilization of HLA-C*14:02 or HLA-C*14:03 on RMA-S cells, likely reflecting a low affinity of HLA-C*14 binding. In a previous study where LC-MS/MS-based immunopeptidome profiling followed by *in vitro* peptide-HLA binding analysis was used to identify HLA-C*12:02-restricted HIV-1 peptides, low-affinity peptides were not found to have elicited T cell responses during natural HIV-1 infection (28). Here, we found that a threshold binding affinity (which corresponded to stabilization of HLA-C*14 expression in the presence of 100 μM peptide at ≥40% of the maximum levels achieved at 26°C in our assays) appeared to be necessary for priming of peptide-specific T cell responses in natural infection (Fig. 3B). In many studies, peptide binding predictions generated using *in silico* methods (such as NetMHCpan) are employed to identify peptides that may putatively constitute novel epitopes (36–40). Importantly, we found mismatches between these *in silico*-predicted binding scores and the binding affinities of peptides determined by *in vitro* experimental analysis. For example, although Pol FF8 and Pol VYL9 were predicted as high-affinity HLA-C*14-binding peptides by *in silico* methods (data not shown), their relative *in vitro* binding affinities were low; moreover, T cell responses were not detected to these peptides. As *in silico* methods for peptide binding prediction are trained on existing data, they frequently perform less well for poorly analyzed HLA alleles such as HLA-C. Our findings highlight the utility of exploiting relative binding affinity measurements from *in vitro* peptide HLA stabilization assays performed with RMA-S cells expressing a single HLA allele as useful index to predict T cell epitopes.

We identified 6 HLA-C*14:02/C*14:03 epitopes in the present study. Five of these peptides were detected by LC-MS/MS in HLA-I eluates from both HLA-C*14:02^+^ and HLA-C*14:03^+^ .221 cells infected with HIV-1, and T cells specific for all 6 epitopes effectively recognized both HIV-1-infected HLA-C*14:02^+^ and HLA-C*14:03^+^ .221 cells. These findings suggest that these 6 peptides are efficiently presented by both HLA-C*14:02 and HLA-C*14:03 molecules in HIV-1-infected cells. Responses to two of these peptides, Gag LY9 and Nef YT9, were identified in a previous study that analyzed T cell responses in both HLA-C*14^+^ and HLA-C*14^−^ individuals by IFN-γ ELISPOT assay and predicted HLA-C*14-restricted epitopes from the results obtained (41). In the present study, we definitively demonstrated that the Gag LY9 and Nef YT9 peptides and 4 additional peptides are HLA-C*14:02/C*14:03 epitopes by showing HLA-C*14-restricted recognition of both peptide-pulsed and HIV-1-infected cells by peptide-specific T cells. Gag LL8-5V7T (LYNTVATL) was previously identified as an HLA-C*14-restricted HIV-1 epitope in a volunteer enrolled in an HIV vaccine trial, where T cell recognition was assessed using a peptide-titrated IFN-γ ELISPOT assay (30). Since the sequence of the reported Gag LL8 epitope differed from that in the NL4-3 strain (LYNTIAVL), we investigated T cell responses to NL4-3-derived Gag LL8 peptide. Although this peptide was detected in HLA-I eluates from both NL4-3-infected HLA-C*14:02^+^ and -C*14:03^+^ cells, no strong T cell responses to this peptide were observed in a cohort of 20 HLA-C*14^+^ HIV-1-infected individuals, although a weak response to Gag LL8-2F5V (LFNTVAVL) was observed in one individual. In contrast, we found relatively strong responses to Gag LY9 in 11 of 60 HLA-C*14^+^ Japanese individuals. Thus, although Gag LL8 is included in Gag LY9, our findings suggest that LL8 is not an epitope, but the 9-mer peptide is recognized as a T cell epitope in Japanese HIV-infected individuals.

HLA-C*14:02 and -C*14:03 differ by only a single substitution at position 21, which lies outside the peptide-binding groove (42). This position is located at edge of the beta-sheet structure, suggesting that it is unlikely to be directly involved in the binding of peptides or peptide-HLA recognition by the T-cell receptor (TCR). We demonstrated in the present study that the overall diversity of the peptides eluted, the peptide length distribution, and the peptide binding motif were all very similar between HLA-C*14:02 and -C*14:03. These findings support the hypothesis that the single amino acid substitution at position 21 does not critically affect the nature of the peptides bound. However, in peptide-HLA stabilization experiments performed with 6 epitope peptides, we found that all 6 peptides had significantly lower relative affinities of binding to HLA-C*14:03 than HLA-C*14:02, suggesting that peptide–HLA-C*14 binding to form a stable complex is influenced by the substitution at position 21. Extrapolating from the crystal structure of HLA-C*03:04 (43) (as the structure of HLA-C*14 has not yet been analyzed), although R21(HLA-C*14:02)/H21(HLA-C*14:03) is located distantly from the peptide binding groove, position 22 (HLA-C*14:02 and -C*14:03; F) next to R21/H21 likely interacts with position 9 (HLA-C*14:02 and -C*14:03; S). Position 9 is located on the bottom of the groove and forms part of the C pocket; hence, it can influence peptide binding (44). We therefore speculate that the difference between R21 and H21 may indirectly impact the conformation and/or flexibility of bound peptides and thus influence the stability of the peptide-HLA complex and affinity of peptide binding.

Epitope-specific T cells were able to recognize both .221-C1402 and .221-C1403 cells pulsed with their cognate peptides or infected with HIV-1. Comparison of differences in the efficiency of T cell recognition of peptide-pulsed cells expressing the two HLA-C alleles was confounded by differences in the HLA-C*14 expression levels on the transfected .221-C14 cell lines employed in our studies, but as no substantial differences were observed in recognition of the two lines despite the higher HLA-C*14 expression on the .221-C1403 than the .221-C1402 cells, it is possible that the lower stability of peptide-HLA-C*14:03 complexes does influence target cell recognition by epitope-specific T cells. Consistent with this, analysis of T cell responses to the 6 epitopes in HIV-1-infected individuals having HLA-C*14:02 or HLA-C*14:03 showed that epitope-specific T cell responses were typically higher in the former group, with the magnitude of T cell responses to Pol KM9 and Nef epitope peptides in HLA-C*14:02^+^ individuals being significantly higher than those in HLA-C*14:03^+^ subjects. The mechanism(s) underlying this difference are not clear, but lower levels of antigen presentation in the context of HLA-C*14:03 may have reduced the induction and/or expansion of T cells specific for these HLA-C*14 epitopes. It was not possible to address whether there are differences in the level of total *ex vivo* expression of HLA-C*14 in HLA-C*14:02^+^ and HLA-C*14:03^+^ individuals due to a lack of HLA-C*14-specific antibodies, but such differences and associated differences in levels of antigen presentation could underlie the disparity observed in HIV-1-specific T cell responses in individuals with these HLA-C*14 subtypes.

A recent study reported that peptide-HLA stabilization is an important determinant of CD8 T cell response immunodominance and HIV-1 control (45). We found no difference in pVL between individuals classified as responders and nonresponders to the 6 viral epitopes identified (data not shown). This potentially suggests that HLA-C*14-restricted T cell responses are not major contributors to the overall efficiency of viral control in HIV-1-infected individuals, although analysis of more individuals is required to draw conclusions. However, in a previous study, we showed that HLA-C*14:03^+^ KIR-2DL2^+^ individuals had significantly lower pVL than those having either HLA-C*14:03 or KIR-2DL2 (15), suggesting that in contrast to T cells, NK cells play a substantial role in reducing pVL in HLA-C*14:03^+^ individuals. Together, these findings raise the hypothesis that the two HLA-C*14 subtypes may offer different advantages in HIV-1 control, with HLA-C*14:02’s superior peptide binding capacity facilitating induction/expansion of HIV-specific CD8 T cell responses but simultaneously delivering stronger inhibitory signals to KIR-2DL2^+^ NK cells, HIV-1 control by which is favored in the context of HLA-C*14:03, whose expression is less well stabilized by peptide binding.

In summary, the present study identified 6 novel HLA-C*14-restricted HIV-1 epitopes using a LC-MS/MS-based approach coupled with peptide binding analysis to select higher-affinity peptides for T cell response screening. Notably, the HIV-1 epitope peptides defined stabilized cell surface expression of HLA-C*14:02 significantly better than HLA-C*14:03, and higher-magnitude T cell responses to the Pol KM9 and Nef epitope peptides were observed in HLA-C*14:02^+^ than in HLA-C*14:03^+^ individuals. These findings demonstrate that an HLA-I polymorphism outside the peptide binding groove can have a significant impact on induction/expansion of pathogen-specific CD8 T cell responses and suggest that in the example studied here, the mechanism involved is likely to entail an indirect effect of the HLA-C*14:02/03 position 21 R/H residue on peptide-HLA-C*14 complex stability. The role of this polymorphism in determining the balance between T cell- and NK cell-mediated pathogen control in infections with other clinically important viruses, including severe acute respiratory syndrome coronavirus 2 (SARS-CoV-2), would be of interest to address in future work.

## MATERIALS AND METHODS

### Ethics statement.

All treatment-naive Japanese adult individuals chronically infected with HIV-1 subtype B were recruited from the National Center for Global Health and Medicine. The study was approved by the ethics committees of Kumamoto University (RINRI-1340 and GENOME-342) and the National Center for Global Health and Medicine (NCGM-A-000172-01). Written informed consent was obtained from all individuals for the collection of blood and subsequent analysis according to the Declaration of Helsinki.

### HLA genotyping.

HLA-A, -B, and -C genotypes were identified by the Luminex microbead method (Luminex 100 System; Luminex) and are reported according to the nomenclature of the HLA Dictionary 200432 at the NPO HLA laboratory (Japan).

### Cell lines.

The HLA class Ia-deficient 721.221 cell line expressing CD4 and transfected with HLA-C*14:02 (.221-C1402) and C*14:03 (.221-C1403) was generated in a previous study (15). The TAP2-deficient mouse RMA-S cell lines expressing HLA-C*14:02 (RMA-S-C1402) and HLA-C*14:03 (RMA-S-C1403) were generated as previously described (15). Both cell lines were cultured in RPMI 1640 medium (Thermo Fisher) containing 10% fetal calf serum (FCS) and 0.15 mg/mL hygromycin B (Calbiochem).

### HIV-1 NL4-3 infection.

.221-C1402 and .221-C1403 cells were infected with HIV-1 NL4-3 in a low volume of RPMI 1640 medium (Thermo Fisher) containing 10% fetal bovine serum (FBS), 2 mM l-glutamine, 100 U/mL penicillin, 100 μg/mL streptomycin, and 10 mM HEPES (R10). Fifty microliters of NL4-3 stock virus (Reverse transcriptase activity (RT) value = 4 × 10^2^ ng/mL) was added to 8 × 10^6^ .221-C1402 cells or .221-C1403, and the cells were then incubated for 1.5 h at 37°C, after which 20 mL of R10 was added and the cells were cultured overnight. At day 1 postinfection, a further 20 mL R10 was added, and cells were split (1:2) on day 2. On day 3 postinfection, the proportion of cells infected with HIV-1 was determined by intracellular p24 staining, as previously described (46). Infected flasks were then harvested for immunoprecipitation of HLA-peptide complexes.

### HLA class I immunoprecipitation.

.221-C1402 cells or .221-C1403 cells infected with NL4-3 (1.5 × 10^8^ to 2 × 10^8^ cells) were harvested, washed in phosphate-buffered saline (PBS), and lysed in 5 mL of lysis buffer (1% IGEPAL 630, 300 mM NaCl, 100 mM Tris [pH 8.0], plus protease inhibitors) at 4°C for 45 min. Two centrifugation steps (2,000 × *g* for 10 min followed by 20,000 × *g* for 30 min at 4°C) were next employed to clear the lysates of cell debris prior to overnight capture of HLA-peptide complexes on W6/32-coated protein A‐Sepharose beads. W6/32-bound HLA-peptide complexes were sequentially washed with 10 to 20 mL of wash buffer 1 (0.005% IGEPAL, 50 mM Tris [pH 8.0], 150 mM NaCl, 5 mM EDTA), wash buffer 2 (50 mM Tris [pH 8.0], 150 mM NaCl), wash buffer 3 (50 mM Tris [pH 8.0], 400 mM NaCl), and finally wash buffer 4 (50 mM Tris [pH 8.0]) under gravity flow in Econo-Column glass chromatography columns (Bio-Rad). Peptide-HLA complexes were eluted from the beads with 5 mL of 10% acetic acid and, after drying under vacuum, were loaded onto a 4.6- by 50-mm ProSwift RP-1S column (Thermo Fisher Scientific) and eluted from an Ultimate 3000 HPLC system (Thermo Scientific) using a 500-μL/min flow rate over 10 min from 2% to 34% buffer B (0.1% trifluoroacetic acid [TFA] in acetonitrile) in buffer A (0.1% TFA in water). Alternate fractions were pooled to give two final peptide fractions that were dried for LC-MS/MS analysis.

### LC-MS/MS.

Each HPLC-eluted sample was resuspended in 20 μL loading buffer, and 9 μL of it was injected onto a 3-μm-particle-size 0.075-mm by 150-mm Acclaim PepMap RSLC C_18_ column and further loaded onto a 2-μm-particle-size, 75-μm by 50-cm Acclaim PepMap RSLC C_18_ column using an Ultimate 3000 nUPLC system (Thermo Scientific). A linear gradient of 3 to 25% buffer B (0.1% formic acid, 5% dimethyl sulfoxide [DMSO] in acetonitrile) in buffer A (0.1% formic acid, 5% DMSO in water) was applied over 1 h to elute peptides from the column (flow rate of 250 μL/min). Peptides were introduced, using an Easy-Spray source at 2,000 V at 40°C, into a Fusion Lumos mass spectrometer (Thermo Scientific). The ion transfer tube temperature was set to 305°C. Full MS spectra were recorded from 300 to 1,500 *m/z* in the Orbitrap at 120,000 resolution with an AGC target of 400,000. Precursors were selected in top-speed mode within a 2-s cycle time (accumulation time of 120 ms) and an isolation width of 1.2 atomic mass units (amu) for fragmentation. Higher-energy collisional dissociation (HCD) with a collision energy setting of 28 was performed on the peptides with a charge state of 2 to 4, while a higher collision energy of 32 was applied to singly charged precursor ions that were selected with lower priority. MS resolution was set at 120,000 and MS2 resolution at 30,000. All fragmented precursor ions were actively excluded from repeated selection for 30 s.

### Analysis of LC-MS/MS data sets.

The analysis of all LC-MS/MS data sets (.raw files) was performed using PEAKS v8.0 (Bioinformatic Solutions) software. No enzyme was specified during the peptide spectral matching, and mass tolerance settings of 5 ppm (for precursor ions) and 0.03 Da (for fragment ions) were used. Spectral sequence annotation was performed against the annotated Homo sapiens Swiss-Prot database appended with a 6-frame translation of the HIV-1 NL4-3 genome. A false discovery rate of 5% was set using a parallel decoy database search. Shannon sequence logos for all unique database-matched (Homo sapiens and HIV) 8-mer to 11-mer peptides were produced using the online tool Seq2logo v2 (47).

### HLA stabilization assay.

The relative affinity of peptide binding to HLA-C*14:02 and -C*14:03 was examined using RMA-S-C1402 and RMA-S-C1403 cells as previously described (15). Briefly, RMA-S cells were cultured at 26°C for 16 h, pulsed with peptides at 26°C for 1 h, and subsequently incubated at 37°C for 3 h. Staining of cell surface HLA-C molecules was performed with the HLA-C-reactive monoclonal antibody (MAb) DT-9 (48), a gift from Mary Carrington, NIH, and fluorescein isothiocyanate (FITC)-conjugated sheep anti-mouse IgG (Jackson ImmunoResearch). Staining data were acquired on a FACSCanto II instrument (BD Biosciences) and analyzed using FlowJo 10.7.1 software. Relative HLA expression was calculated as the ratio of the mean fluorescence intensity (MFI) of peptide-pulsed RMA-S-C1402 cells or RMA-S-C1403 cells to that of control (non-peptide-pulsed) cells kept at 26°C, multiplied by 100 to express the values for peptide-pulsed cells as a percentage of that for the control cells at 26°C.

### Peptide-HLA complex dissociation assay.

A peptide-HLA complex dissociation assay was performed as previously described (49). The stability of complexes formed between the 6 peptides and HLA-C*14:02 or -C*14:03 molecules was determined using RMA-S-C1402 and RMA-S-C1403 cells. The cells were cultured at 26°C for 16 h and then incubated at 26°C a further 1 h in the presence of 100 μM peptide or medium only as a negative control. The cells then warmed to 37°C, incubated for 3 h in the continued presence of peptide (or medium only), washed three times with cold culture medium to remove excess external peptide, and cultured in medium without peptide. After 0, 1, 2, and 4 h, samples were taken, washed twice, immediately stained with HLA-C-reactive MAb DT-9 for 30 min, washed, and stained with FITC-conjugated sheep anti-mouse IgG (Jackson ImmunoResearch). Staining data were acquired on a FACSCanto II instrument (BD Biosciences) and analyzed using FlowJo 10.7.1 software. The decay of HLA-C*14:02–peptide and HLA-C*14:03–peptide complexes was determined as the percent MFI remaining: (MFI_time__(+peptide)_ − MFI_time__(−peptide)_)/(MFI_time__=0(+peptide)_ − MFI_time__=0 (−peptide)_) × 100.

### ELISPOT assay.

PBMCs were separated from whole blood and stored at −80°C. ELISPOT assays were performed as previously described (13). Briefly, 1 × 10^5^ PBMCs from HIV-1-infected individuals and a 1 μM concentration of each HLA-C*14:02- and -C*14:03-eluted HIV-1 peptide were added to 96-well polyvinylidene plates (Millipore) that had been coated overnight with 5 μg/mL anti-IFN-γ MAb 1-D1K (Mabtech). The plates were incubated for 16 h at 37°C in 5% CO_2_, and then the cells were stained as previously described in detail (13). The spots were counted with an Eliphoto-Counter instrument (Minerva Teck). The number of spots was calculated per 10^6^ PBMCs; 100 spots/10^6^ PBMCs was defined as a positive response, as described previously (13).

### HIV-1-specific T cell expansion and intracellular cytokine staining assay.

PBMCs from HIV-1-infected individuals were stimulated with 1 μM concentrations of the corresponding peptides and then cultured for 2 weeks in RPMI 1640 medium (Thermo Fisher) containing 10% FCS, 20 ng/mL of human recombinant interleukin 2 (rIL-2) (ProSpec), 1× minimal essential medium (MEM) nonessential amino acid solution (Gibco), and 1 mM sodium pyruvate solution (Gibco). Following T cell expansion, .221-C1402 cells or .221-C1403 cells prepulsed with each HIV-1 peptide or .221-C1402 cells or .221-C1403 cells infected with HIV-1 NL4-3 were added to a 96-well plate together with bulk-cultured T cells, and the cells were incubated for 4 h at 37°C with brefeldin A (10 g/mL). The cells were then stained with Pacific blue (PB)-labeled anti-CD3 MAb (BioLegend), FITC-labeled anti-CD19 MAb (BioLegend), allophycocyanin (APC)-labeled anti-CD8 MAb (BioLegend), and a LIVE/DEAD fixable near-infrared (IR) dead-cell staining kit (Invitrogen) and subsequently fixed with 4% paraformaldehyde and incubated in permeabilization buffer (0.1% saponin–5% FBS–PBS). Thereafter, the cells were stained with phycoerythrin (PE)-labeled anti-IFN-γ MAb (BioLegend). Staining data were acquired on a FACSCanto II instrument (BD Biosciences) and analyzed using FlowJo 10.7.1 software.

### Statistical analyses.

The statistical significances of differences in peptide binding affinities and surface stability between HLA-C*14:02 and HLA-C*14:03 were calculated using an unpaired *t* test, and *P* values were corrected to account for multiple testing. Both *P* values and false discovery rate (FDR)-corrected *q* values are reported. Correlations between HLA-C*14:02 and HLA-C*14:03 T cell recognition and peptide binding ability were statistically analyzed using Pearson's correlation coefficient. *P* values of <0.05 were considered statistically significant. Statistical significances of differences in binding ability or the magnitude of T cell responses between HLA-C*14:02^+^ and HLA-C*14:03^+^ individuals were calculated using the Mann-Whitney test. The statistical differences of binding affinities between HLA-C*14:02 and HLA-C*14:03 at each concentration of peptides were calculated using an unpaired *t* test.
